# A Puzzle for the Water Scientists

**Published:** 1881-10

**Authors:** 


### A PUZZLE FOR THE WATER SCIENTISTS

Has lately been propounded in one of the Liverpool papers. "A Vicar," having taken up his residence in the country, was greatly alarmed to find the people resorting to the ditches to eke out their supply of drinking water. The luxury of spring water was unattainable, and the only resource was rain water stored in tanks or caught in tubs, supplemented by the friendly ditch, from whence the people were content to drink, not only when the water looked tolerably fair to the eye, but sometimes when it was positively green. Contrary to all that "A Vicar" had been led to expect under such circumstances, the people were healthy and long-lived. "The deaths of children," he found, "were not six in a thousand." The reverend gentleman was, therefore, constrained to write as follows: "Whatever may be said in explanation, the case is this: The water drunk by the inhabitants of this locality is rain water and ditch water, and yet there are many very old persons, and very few deaths, and very little disease." Mr. Alfred Smetham, F.C.S., a member of the Society of Public Analysts, considers the statement of "A Vicar" to be one which is "calculated to mislead." The point raised by the clergyman was this: That, according to the usual theory, the people must have "good, wholesome water," or sickness and death would prevail. Was ditch water good and wholesome? Mr. Smetham bases the quality of the water on the presence or absence of "disease germs," and says that the "green matter" in the ditch water may be no more injurious than an equal quantity of watercress. "Water from a well-kept ditch," he observes, "is safer than water taken from a well in close proximity to a cesspool." No doubt Mr. Smetham is so far right. But if a "well-kept ditch is to be tolerated, what does Mr. Smetham think of the Thames at Hampton, and the character of the water supply which has been drawn through the London filter-beds? If the persistent healthfulness of the population referred to by "A Vicar" is consistent with the drinking of green ditch water, there is surely a pretty good prospect for the inhabitants of the Metropolis. If the London Water Companies were to supply ditch water on the particular day in the month when Dr. Frankland takes his samples, we might look for an analytical report which would almost frighten the Metropolis into a fever. We believe it very often happens that people who are extremely fastidious about the London water supply will gulp down almost anything that looks like a decent glass of water when they are in the country. The proverb which speaks of the gnat and the camel is not wholly inapplicable in these cases.—*The Journal of Gas Lighting*.

